# Brain connectivity and its relation to cognitive function in patients with post-COVID 19 condition after mild infection

**DOI:** 10.1038/s41598-026-41665-2

**Published:** 2026-03-03

**Authors:** Stina Hedström, Jonas Stenberg, Kristian Borg, Sonia Miri Hedberg, Tobias Granberg, Alexandra Gyllenberg, Sven Pettersson, Hadrien Van Loo, Marika C. Möller, Love Engström Nordin

**Affiliations:** 1 Department of Clinical Sciences, Danderyd Hospital, Karolinska Institutet, Stockholm, Sweden; 2 Department of Rehabilitation Medicine, Danderyd University Hospital, Stockholm, Sweden; 3 Department of Clinical Neuroscience, Karolinska Institutet, Stockholm, Sweden; 4 Department of Neuroradiology, Karolinska University Hospital, Stockholm, Sweden; 5 Department of Nuclear Medicine and Hospital Physics, Karolinska University Hospital, Stockholm, Sweden; 6 Department of Neurobiology, Care Sciences and Society (NVS), Karolinska Institutet, Stockholm, Sweden

**Keywords:** Post COVID-19 condition, Brain function, fMRI, Functional connectivity, Cognitive symptoms, Diseases, Neurology, Neuroscience

## Abstract

Neurological symptoms are common in post-COVID-19 condition (PCC) and have been linked to underlying brain alterations. However, in individuals with PCC following a mild infection without hospitalization, such alterations are rarely detected using conventional neuroimaging techniques. This study aims to investigate brain connectivity in patients with PCC with cognitive symptoms after mild COVID-19 infection, using resting-state functional magnetic resonance imaging (rs-fMRI). Additional aims were to explore associations between brain connectivity, neuropsychological performance, and self-reported fatigue and emotional status. Patients with PCC (*n* = 22) and lasting cognitive symptoms and fatigue were consecutively recruited from a regional rehabilitation unit and compared with a convenience sample of non-symptomatic controls (*n* = 19). The assessments were conducted on average 32 months post-infection and included 3 Tesla rs-fMRI, neuropsychological testing, and self-report measures of fatigue (MFI-20), anxiety, and depression (HADS). Patients with PCC had elevated functional connectivity in brain regions associated with the default mode network (DMN) compared to controls. No significant correlations were found between functional connectivity, neuropsychological test performance, fatigue, anxiety, or depression. Our findings suggest persistent alterations in DMN connectivity in PCC with cognitive symptoms and fatigue, underscoring the need for continued larger studies on brain functioning in this patient group.

**Clinical trial registration**: No. NCT06042530.

**Supplementary Information:**

The online version contains supplementary material available at 10.1038/s41598-026-41665-2.

## Introduction

The brain correlates underlying cognitive deficits and fatigue in Post COVID-19 condition (PCC) is still largely unknown^1^. However, 1.8% of people once infected with SARS-CoV-2 report symptoms for 12 weeks or more^2^, and PCC has been rated as a global health burden^3^. As part of the condition, cognitive difficulties and fatigue are common, and cognitive deficits have been reported in domains such as attention, memory, processing speed and executive function, also after an initial mild infection^4,5^. A striking feature of PCC is functional impairment^6^, and evidence has linked cognitive deficits in PCC to underlying brain changes^7^. People in working ages (35–65 years) are most frequently affected^2^, and given the current uncertainty in healthcare regarding optimal management of cognitive impairments, there is an urgent need to further map PCC-related effects on the brain.

Research has linked structural brain changes, such as grey and white matter alterations, to cognitive decline in PCC^8–11^, but these are seldom detected in clinical setting, when using conventional neuroimaging techniques such as computed tomography or magnetic resonance imaging (MRI). Functional MRI (fMRI) may be more sensitive to PCC-related changes since it can map brain activity patterns across brain regions^12^. However, studies of the brains functional capacity in PCC are spare, but resting state fMRI (rs-fMRI) has emerged as a valuable tool in PCC. In a study using multimodal MRI in patients with PCC after mild infection, only rs-fMRI captured differences in brain activity between patients and healthy controls, supporting the method’s sensitivity in the patient group^13^. rs-fMRI has also revealed changes in brain functioning in conditions with overlapping symptoms to PCC, such as mild traumatic brain injury and chronic pain^14,15^.

Previous research on brain functioning points to disruptions in the brain’s functional connectivity, with both increases and decreases in connectivity among people with PCC^8,16–19^, but as functional methods vary between studies, PCC is seldom clearly defined, and the severity in the initial infection vary, comparison between studies is difficult. Though, in a sample of participants with PCC as defined by WHO-criteria with various severity of initial disease, patients showed increases in intra-network functional connectivity in the default mode, salience, executive control, auditory and basal ganglia networks, compared to controls when examined with rs-fMRI^20^.

However, very few studies investigate brain functioning in patients with PCC who initially had a mild infection, which is especially important as this group represent the majority of people living with PCC^6,21,22^. Also, studies on patients with an initial mild infection help us separate potential brain effects of the virus itself from the effects of treatment on an intensive care unit, which in itself has been linked to cognitive and psychiatric disruptions^23^. Studies comparing patients with PCC with an initial mild disease with healthy controls, using rs-fMRI, show mixed results regarding network alterations. For example, Burgenberg, et al. pointed out both increases and decreases in patients´ functional connectivity in widespread network connections 7 months post-infection^24^, while Fineschi, et al. showed functional connectivity alterations limited to a few brain regions 20 months after infection, including higher connectivity the middle frontal gyrus, connecting voluntary and automatic attention networks^13^. As today, very few studies on patients with PCC after a mild infection measure the long-term effects on brain functioning as well as why conclusions on more persistent changes are uncertain. Further, there is a still need to define how neuroimaging findings relate to PCC-related cognitive difficulties, as findings across studies are inconclusive. For example, elevated brain functional connectivity has been associated with both self-reported memory difficulties and fatigue^13,20,24^ and a *higher* performance on cognitive tests, or failed to show significant relations with test performance^20,25^.

This study therefore aimed to: (1) Compare brain connectivity between patients with PCC following a verified mild SARS-CoV-2 infection and non-symptomatic controls, using rs-fMRI and (2) Evaluate the relation of functional brain alterations to the performance on neuropsychological tests and self-reports of fatigue, anxiety and depression.

## Methods

### Participants and procedure

Patients were consecutively enrolled at the outpatient Cognitive Post COVID-19 Clinic at Danderyd University Hospital Stockholm, Sweden, where they were referred due to long-lasting cognitive PCC symptoms negatively affecting work and daily activities. The majority of the referrals to the clinic came from primary care units. All patients first underwent a medical examination to confirm the PCC diagnosis according to WHO criteria^26^, followed by an extensive neuropsychological assessment.

Patients were recruited at the time of their clinical neuropsychological assessment. The control group consisted of a convenience sample recruited trough advertisement in a university setting and among hospital staff. Controls were recruited with the intention to match the patient group with regards to age, sex, and educational level. Because population-wide testing was not conducted in Sweden during the pandemic, it was not possible to either confirm or rule out whether the control participants had previously experienced a SARS-CoV-2 infection. Instead, during the inclusion of controls, efforts were made to ensure that they had not experienced prolonged symptoms after a previous infection. If so, they were excluded. Recruitment took place between April 2022 and January 2024.

The study was approved by the Swedish Ethical Review Authority (reference numbers 2021–03907 and 2022-01215-02) and was conducted in accordance with the Declaration of Helsinki. All study participants gave written informed consent after receiving oral and written information about the study. The study was pre-registered in Clinical Trials (No. NCT06042530).

### Inclusion and exclusion criteria

The inclusion criteria for patients were: (1) Mild SARS-CoV-2 infection without a need or hospitalization confirmed via polymerase chain reaction test or antibody testing; (2) PCC diagnosis according to WHO-criteria; (3) Self-reported cognitive symptoms persisting ≥ 3 months; (4) Age 25–55 years (to minimize the risk of the brain not being fully developed or age-related brain changes).

The exclusion criteria for patients were: (1) Dominant recurring and/or fluctuating infection symptoms, circulatory, respiratory, or cardiological problems; (2) Severe ongoing mental illness comorbidity with pronounced cognitive impairment; (3) Cognitive difficulties primarily related to neurodegenerative disease, addiction, or dementia; (4) Neuropsychiatric disorders such as diagnosed ADHD or autism; (5) Severe visual impairment (6) Non-proficiency in the Swedish language (since self-reports and tests requires good language skills in the language they are administered in); (7) Left-handedness (to increase the uniform topological lateralization in the cohort); (8) MRI contraindications. The same exclusion criteria were applied for controls. In addition, controls did not have previous or ongoing PCC. Before the brain MRI examination, all participants were screened for MRI contraindications such as implanted metals, pregnancy, and strong discomfort in cramped spaces.

#### Neuropsychological assessment

Patients and controls were assessed with the same self-report questionnaires and neuropsychological test battery. The majority of participants were assessed by licensed psychologists, but some controls were assessed by psychology students. Students were thoroughly informed about test procedures, and students’ adherence to test protocols and test scoring were continuously checked by the licenced psychologists to ensure correct administration.

#### Self-reports

The following scales were used: (1) The Multidimensional Fatigue Inventory-20 (MFI-20), measuring general, physical and mental fatigue and reduced activity and motivation;^27^ (2) The Hospital Anxiety and Depression Scale (HADS), measuring depression- and anxiety symptoms^28^.

#### Neuropsychological tests

Neuropsychological tests were selected based on findings from a larger study conducted by our research group (submitted manuscript), where chosen tests showed the largest differences in test performance between patients and controls.

The tests were (1) Bushke Selective Reminding Test^29^, measuring verbal learning and memory (2) WAIS-IV Coding^30^, measuring processing speed (3) D-KEFS Color-Word Interference Test^31^, measuring attention and executive functioning (4) Ruff 2&7^32^, measuring selective attention. Also, WAIS-IV Matrix^30^ was used as it measures non-verbal logic reasoning and is to be considered robust against cognitive decline. The tests are further described in Supplementary, Table 1 (adapted from a previous submitted manuscript by our research group to Scientific Reports, ID 84de0c45-3f71-4269-a126-50b432e309bc).

**Table 1:** Characteristics of patients with PCC and the control group.

	PCC *n* = 22	Controls *n* = 19	*p*
Age, years			
Mean (SD)	44.2 (7.7)	39.2 (10.1)	
Median (IQR)	44.0 (37.5–51.3)	38.0 (29.0–50.0)	0.129
Sex, female, %	68.2	73.7	0.699
Education, %			
University	68.2	100	0.010
Body mass index			
Mean (SD)	26.7 (4.6)	24.5 (4.1)	
Median (IQR)	26.1 (23.7–27.8)	23.8 (22.3–25.8)	0.044
Year of infection, %		N/A	
2020	54.6		
2021	40.9		
2022	4.6		
Time between infection and imaging, months		N/A	
Mean (SD)	32.4 (7.1)		
Median (IQR)	33.5 (25.0-36.5)		
Sick leave, %		N/A	
25–99	13.6		
100	31.8		

Note. IQR, interquartile range; NP-assessment, neuropsychological assessment; PCC, post COVID-19 condition; SD, standard deviation. Mann-Whitney U tests and Chi-square tests were used for group comparisons.

#### Image acquisition

Brain MRI examinations took place at Karolinska University Hospital, Huddinge, Sweden, with a median time of 55 days (IQR 44.25–63.25) after the neuropsychological assessment. Participants were examined with a 3 Tesla Siemens MAGNETOM Prisma^Fit^ system (Siemens Healthineers, Erlangen, Germany) equipped with a 64-channel receive-only head coil. Conventional clinical sequences were acquired, including 3D T1-weighted Magnetization-Prepared Rapid Gradient-Echo, T2-weighted Fluid-Attenuated Inversion Recovery and susceptibility-weighted imaging. During a 20-minute-long cognitive task, measuring sustained vigilance using a reaction-time task (PVT), Pseudo-Continuous Arterial Spin Labeling (pCASL) was acquired for brain perfusion. Results from the pCASL measurement are not reported in this study. Blood Oxygenation Level Dependent rs-fMRI was acquired before and after the PVT, where participants were instructed to focus their gaze on a white cross on a black background on the display, and not to think about anything special. These instructions aimed at minimizing the risk of participants falling asleep during the MRI and minimizing the variance of visual stimuli. The rs-fMRI sequences had an echo time of 33 ms and a repetition time of 1000 ms, isotropic resolution of 2 mm^3^, 84 interleaved slices and a field-of-view 220 × 220 mm. Both sequences included 284 measurements, lasting for 5:01 min. The total effective scan time was 45:20 min, where the rs-fMRI lasted in total 10:02 min. The anatomical sequences were screened by a board-certified neuroradiologist for structural pathological findings.

#### Image post processing

The rs-fMRI data were preprocessed using the FMRIB Software Library (FSL 6.0.6.2) (http://www.fmrib.ox.ac.uk/fsl). A high pass filter cutoff of 100 s (0.01 Hz) was applied to remove low frequency, non-neural signals. Intra-modal motion correction was carried out using MCFLIRT, which applies rigid-body transformations to correct head motion in the fMRI time series^33^. Translational and rotational motion parameters were inspected for all participants to ensure that head displacement did not exceed one voxel size (2 mm) since the motion correction applied is sub-optimal for larger motion. No global signal regression (GSR) was performed to avoid the mathematical introduction of artificial anti-correlations.

Non-brain tissue was removed using the Brain Extraction Tool^34^. To improve the signal-to-noise ratio and reduce high-frequency spatial noise, spatial smoothing was applied with a Gaussian kernel of 5 mm full-width at half maximum. The functional data were then spatially normalized to the Montreal Neurological Institute (MNI) standard brain template using a 12-parameter affine transformation with a mutual-information cost function, resampling the data to an isotropic voxel resolution of 4 mm. This normalization process ensures that individual brain images are aligned within a common stereotaxic space, allowing for voxel-wise group-level analyses while preserving anatomical correspondence across subjects.

Independent Component Analysis (ICA) was performed using FSL’s MELODIC (version 3.15) with the multisession temporal concatenation approach^35^. ICA is a data-driven, exploratory technique that separates the fMRI signal into statistically independent spatial and temporal components. It is particularly effective for identifying and removing noise sources, such as those arising from physiological motion (e.g., respiration, cardiac pulsation), and for detecting spontaneous or random neural responses^36^. ICA was run across all participants to achieve robust estimation of group-level components representing common spatial patterns of activity, thereby minimizing group bias. The number of output components was limited to 20 to avoid overfitting. The resulting components were classified manually by considering the spatial maps, time courses and the power spectra of each component. Components identified as noise (e.g., motion, vascular, or CSF artifacts) were excluded from further analysis, effectively serving as an ICA-based denoising step prior to dual regression.

Dual regression analyses were then employed to project these group-level spatial maps back into individual participants’ data in order to assess temporal dynamics and within-group differences^37^. In the first stage, each subject’s 4D dataset was regressed onto the group-average spatial maps to obtain subject-specific time series corresponding to each component. In the second stage, these time series were regressed back into the same 4D dataset to generate subject-specific spatial maps for each group-level component.

Contrast matrices were defined in FSL’s General Linear Model (GLM) to perform two-way analyses of group effects, allowing assessment of differences in functional connectivity between brain networks.

To examine temporal effects, the rs-fMRI data from before and after PVT were included. Separate contrast matrices were generated within the GLM framework to compare each time point in both directions, for patients and controls, respectively. Models were also created to test for group effects using independent variables as regressors, such as the participants performance on neuropsychological tests, self-reports on MFI subscales and HADS anxiety and depression. Group- and time effects were assessed using FSL’s Randomise tool for non-parametric permutation testing^38^(10.000 permutations). Results were thresholded at *p* < 0.05 (family-wise error corrected) using Threshold-Free Cluster Enhancement (TFCE). While a minimum cluster size of 2 contiguous resampled voxels (16 acquired voxels) was used for reporting, the use of TFCE ensures that significance is determined by both local signal intensity and spatial extent without the need for an arbitrary initial cluster-forming threshold.

#### Statistical analyses

To compare groups on age, sex, educational level and body mass index, the Mann–Whitney U-tests was applied on continuous, non-parametric data, and the Chi-square test was used on categorical data. Groups were compared on self-rated fatigue (MFI-20), anxiety and depression (HADS) using the Mann-Whitney U-test.

Neuropsychological test scores were standardized using norms from the test manufacturer before analysis. Regression models were run to evaluate group effects (i.e. PCC vs. non-symptomatic controls). Because of a higher educational level in the control group, educational level was included as a covariate in all models except for when evaluating group performance on the test Ruff2&7, where test manufacturer standardization includes educational level. Main assumptions for regressions (no multicollinearity, normality of residuals, linearity, homoscedasticity) was checked in all models and considered satisfactory. Analyses were done in IBM SPSS, version 29. Missing data was handled by pairwise deletion.

## Results

### Demographic and clinical characteristics

The mean age in the PCC group was 44.2 years. 68% were women and 68% had a university education. (Table 1). There were no significant group differences in age or sex between patients and controls. A significantly larger proportion of controls were higher educated, but patients and controls had very similar scores on estimated premorbid IQ measured by Matrix reasoning (Table 2).

**Table 2:** Neuropsychological test performance in patients with PCC and controls.

Neuropsychological test	PCC (*n* = 22)	Controls (*n* = 19)	Regression results		
			Group effect	CI 95%	*p*
WAIS-IV Matrix Reasoning					
Mean (SD)	12.9 (2.6)	13.3 (3.2)	0.55	-1.35-2.45	0.561
WAIS-IV Coding					
Mean (SD)	10.9 (2.3)	12.2 (2.0)	− 0.83	-2.34-0.69	0.278
D-KEFS Color-Word Inhibition					
Mean (SD)	10.6 (3.1)	11.9 (2.7)	-1.30	-3.39-0.80	0.218
Buschke Selective Reminding Test					
Total recall					
Mean (SD)	45.9 (10.4)	43.7 (14.5)	1.92	-6.91-10.74	0.663
Delayed recall					
Mean (SD)	40.9 (13.4)	42.8 (16.2)	− 0.19	-10.56-10.18	0.971
Ruff 2&7					
Automatic speed					
Mean (SD)	48.6 (10.6)	50.6 (9.9)	-2.04	-8.55-4.47	0.530
Controlled speed					
Mean (SD)	44.1 (10.1)	45.9 (10.0)	-1.76	-8.11-4.59	0.579

Note. CI, confidence interval; D-KEFS, Delis–Kaplan Executive Function System; PCC, post COVID-19 condition; SD, standard deviation; WAIS-IV, Wechsler Adult Intelligence Scale-IV.

Test scores were standardised using norms from test manufacturer before regression analysis, corresponding scaled scores (mean 10, SD 3) or T-scores (mean 50, SD 10). Educational level was adjusted for in analysis except for Ruff2&7 where educational level is included in test manufacturer standardization.

Over half of the patients had their initial infection in 2020. When brain MRI was performed, patients had experienced symptoms for an average of 32 months, and 45% were on sick leave to some extent.

Patients rated high self-perceived fatigue on MFI, with the highest symptom burden on subscales measuring general and physical fatigue. Patients mean levels of depression on HADS were in the mild range, whereas their ratings on HADS anxiety were not elevated according to instrument guidelines. On self-reports, patients consistently rated higher symptom burden than controls (Table 3).

**Table 3:** Self-reported depression, anxiety and fatigue in patients with PCC and controls.

	PCC	Controls	*p*
HADS Depression			
n	21^1^	19	
Median (IQR)	9.0 (4.5–13.0)	1.0 (0.0–2.0)	< 0.001
HADS Anxiety			
n	21^1^	19	
Median (IQR)	7.0 (4.5–11.0)	2.0 (1.0–5.0)	0.001
MFI-20 General Fatigue			
n	22	19	
Median (IQR)	18.0 (15.6–20.0)	9.0 (6.0–11.0)	< 0.001
MFI-20 Physical Fatigue			
n	22	19	
Median (IQR)	18.5 (13.6–20.0)	6.0 (5.0–7.0)	< 0.001
MFI-20 Reduced Activity			
n	22	19	
Median (IQR)	16.0 (12.8–18.3)	7.0 (5.0–8.0)	< 0.001
MFI-20 Reduced Motivation			
n	22	19	
Median (IQR)	13.0 (9.0-17.3)	5.0 (4.0–6.0)	< 0.001
MFI-20 Mental Fatigue			
n	22	19	
Median (IQR)	16.0 (13.0–18.0)	8.0 (6.0–10.0)	< 0.001

Note. HADS, Hospital Anxiety and Depression Scale; IQR, interquartile range; MFI-20, The Multidimensional Fatigue Inventory; PCC, post COVID-19 condition; SD, standard deviation. Mann-Whitney U tests were used for group comparisons. ^1^One patient had missing values on HADS but completed the other self-reports.

No significant group effects (i.e. patients vs. controls) were found on neuropsychological test performance on the 7 test variables measuring logic reasoning, processing speed, visual attention/inhibition, verbal learning and memory and selective visual attention and speed (Table 2).

Associations between BMI and mean network strength was examined by averaging individual dual-regression Z-scores within the default mode network. No significant correlations were observed within either group.

### Neuroimaging findings

**Fig. 1:**
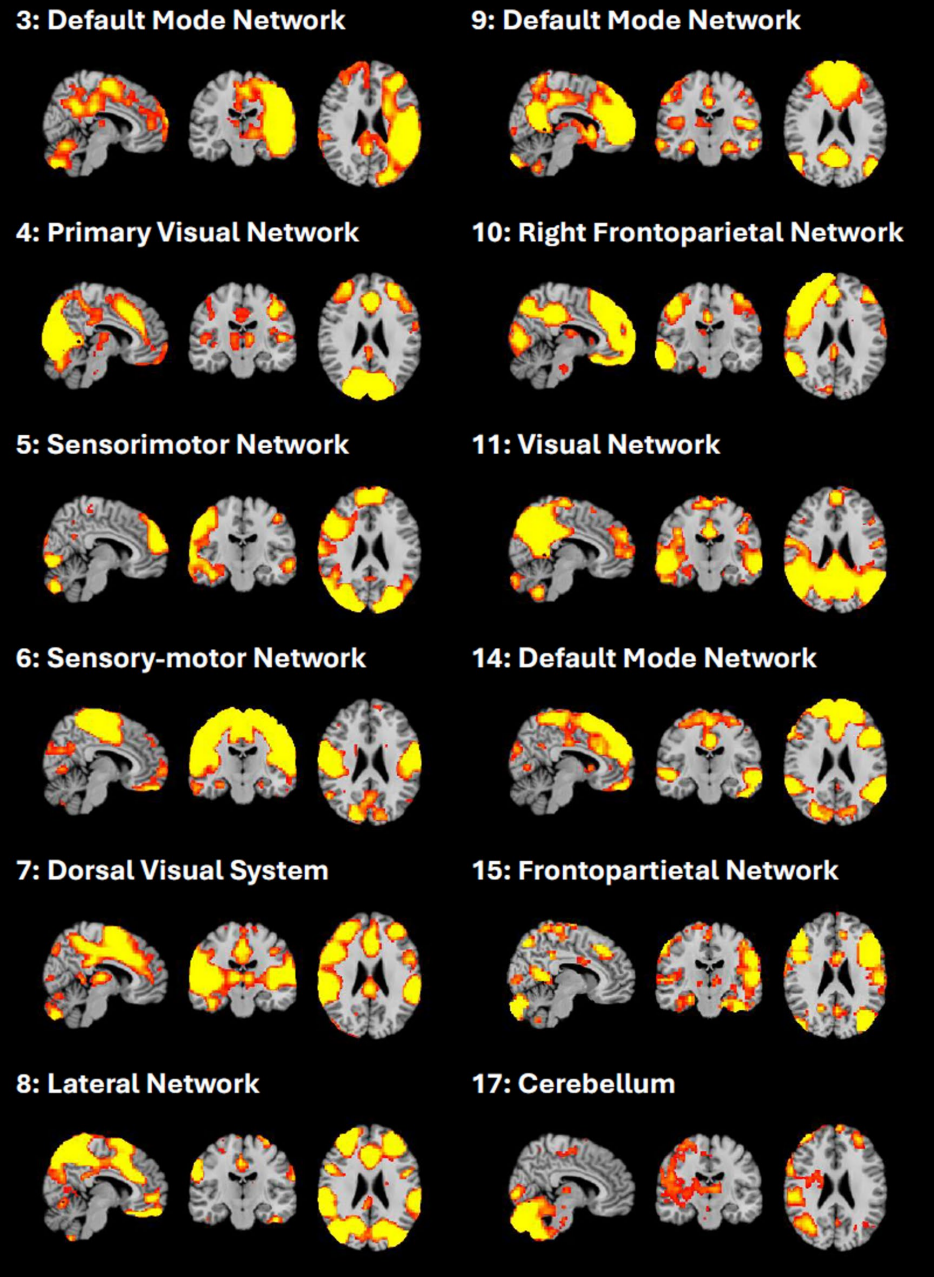
Identified group-level components (IC) from the ICA analysis from manual classification.

The ICA analysis of the rs-fMRI data from both time points (pre- and post-PVT) identified 20 independent components, of which 8 were classified as artifacts—primarily related to motion and cardiac noise—and were therefore excluded from further analysis. Figure 1 shows the remaining 12 networks.

### Differences in resting-state networks

Three controls were excluded after the brain MRI examination. One control was excluded because of motion artefacts (defined as motion larger than one voxel size, 2 mm) and one after expressing sickness symptoms. The third control was excluded because of brain MRI findings consistent with multiple sclerosis. None of the remaining participants (patients *n* = 22, controls *n* = 19, presented in Table 1), had any pathological structural brain abnormalities on the anatomical sequences.

**Fig. 2:**
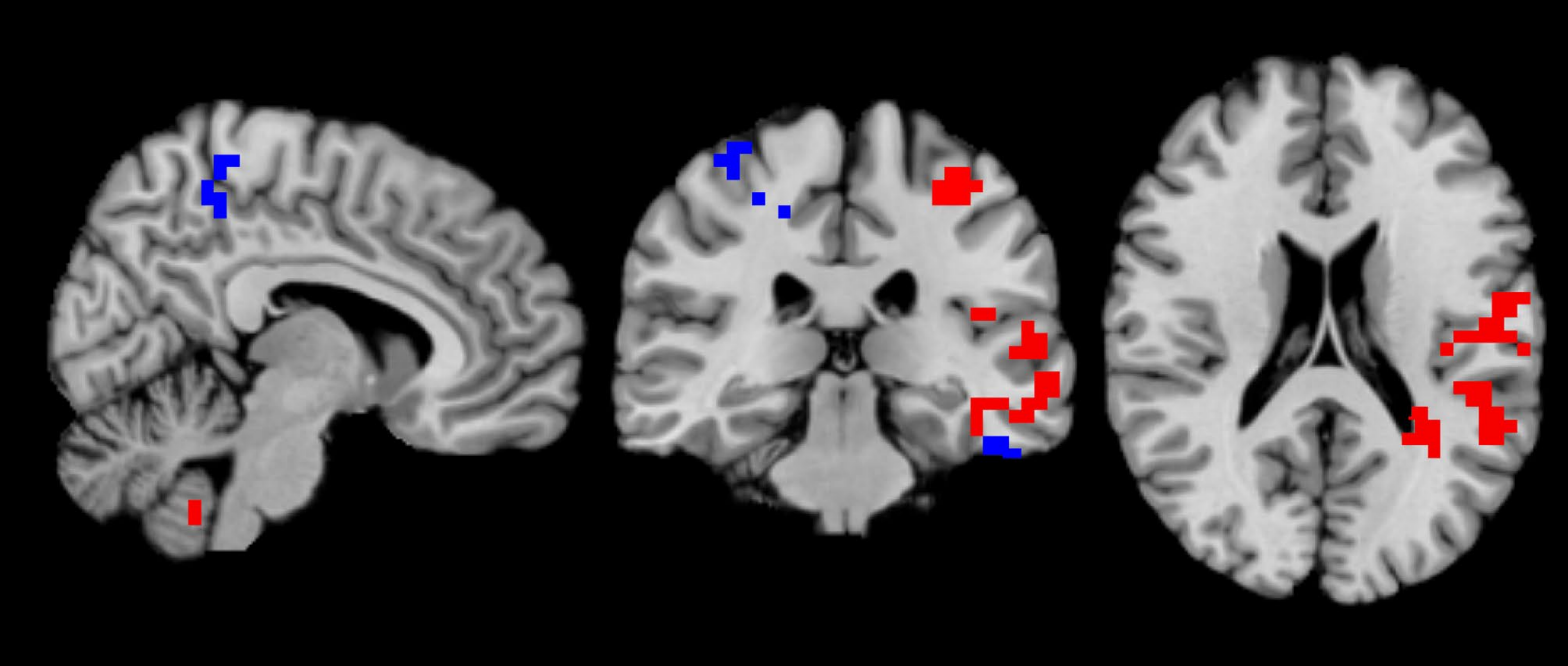
Brain clusters that correlate significantly higher with IC in the patient group than in the control group. Correlations with IC3 are marked with red, and with IC14 are marked with blue.

Group-level dual regression showed significant functional connectivity differences between patients and controls, in brain areas associated with two default mode networks (DMN) (network 3 and 14 in Table 4) before the PVT. In brain clusters showing differences in connectivity between groups, patients consistently had increased connectivity compared to the controls. The majority of significant clusters were located within the DMN (Table 4; Fig. 2, Supplementary Fig. 1) and included the left cerebellum, left postcentral gyrus, left insula lobe, right postcentral gyrus, left fusiform gyrus, left inferior temporal gyrus, left and right middle orbital gyrus, right temporal lobe and right precuneus (marked with an asterisk in Table 4). No significant time effect (before vs. after PVT) was found in any group after controlling for multiple comparisons (p-value set to < 0.025). When comparing patients and controls on data from the resting-state session after the PVT, no significant differences in functional connectivity were found.

**Table 4:** Regions with significantly higher connectivity to group level components for patients compared to healthy controls before PVT.

Brain region	IC	MNI coordinate (x, y, z)	Cluster volume (ml)
L Cerebellum	3	− 34, − 62, − 49	61.504*
L Postcentral Gyrus	3	− 42, − 14, 47	2.944*
L Postcentral Gyrus	3	− 38, − 30, 55	1.728*
L Insula Lobe	3	− 38, 10, − 1	1.344*
L Cerebellum	3	− 54, − 50, − 37	0.192*
R Postcentral Gyrus	14	26, − 38, 51	5.440*
R Middle Orbital Gyrus	14	18, 66, − 13	2.752*
R Precentral Gyrus	14	42, 6, 47	2.496
L Fusiform Gyrus	14	− 42, − 66, − 13	2.048*
L Inferior Temporal Gyrus	14	− 54, − 34, − 29	1.280*
L Middle Occipital Gyrus	14	− 26, − 82, 11	0.768
L Middle Orbital Gyrus	14	− 18, 54, − 17	0.640*
R Middle Orbital Gyrus	14	38, 38, − 13	0.448*
R Inferior Frontal Gyrus	14	42, 30, 3	0.384*
R Temporal Pole	14	50, 14, − 17	0.256*
R Precuneus	14	6, − 42, 59	0.192*
L Precuneus	14	− 14, − 42, 43	0.128

*Clusters overlapping with IC.

Note. IC, independent component; L, left; MNI coordinate, Montreal Neurological Institute coordinate; PVT, Psychomotor Vigilance Task; R, right. p-value for group comparison set to 0.05.

### Correlations between resting-state networks, test-performance and symptom measures

We found no significant correlations between functional connectivity and neuropsychological test performance in neither patients nor controls. Furthermore, we did not find any correlations between functional connectivity and self-reported ratings of fatigue, anxiety, or depression, on any time point in connection with the PVT for neither patients nor controls.

## Discussion

The aim of the study was to compare brain connectivity between patients with mild PCC after a verified SARS-CoV-2 infection with non-symptomatic controls and to evaluate if functional brain connectivity was related to patients’ performance on neuropsychological tests and self-rated fatigue, anxiety and depression. We found significant group differences in resting-state functional connectivity in the DMN, with patients showing increased connectivity before a sustained attention (vigilance) task, but not after the task. No significant associations were found between functional connectivity and neuropsychological test performance and self-ratings of fatigue and emotional symptoms in neither patients nor controls.

Our findings align with previous studies showing increased connectivity in DMN regions in patients with acute COVID-19 compared to controls^16,39^. A rise in intra-network connectivity in multiple brain networks, including the DMN, has also been reported by Carreras-Vidal, et al. in patients with PCC^20^, but in contrast did patients in our study show alterations limited to the DMN. The differences in extent of network alterations might be due to different patient groups, with Carreras-Vidal investigating patients with a varied severity of initial infection, and our sample consisting exclusively of patients with an initial mild infection. Also, the differences in performance on cognitive tests between patients and controls are difficult to compare due to differences in sample sizes, as Carreras-Vidal observed lower performance in their 121 PCC-patients in a variety of tests, while the 22 patients in this study did not differ significantly in test performance compared to controls. However, trends of poorer test performance were evident on the majority of tests, indicating that our lack of significant differences could be related to low power in our study. Still, it is possible that degree of cognitive difficulties between studies are mirrored in the rs-fMRI findings, with more extensive difficulties reflected across multiple networks affecting the brains capacity to engage in goal-oriented cognition, while the symptoms in our sample being more cohesively restricted to the brains capacity to engage in wakeful/restorative rest. In line with this interpretation did Fineschi, et al. show connectivity differences in 36 patients with PCC after mild infection in areas important in convergence of attention networks, in patients scoring significantly lower than controls on a neuropsychological screening tool on attention, language, and memory domains^13^. In a study by Scardua-Silva and coworkers they were not able find any functional connectivity differences between 97 patients and 77 controls in rs-fMRI, when focusing on the posterior cingulate cortex as a core hub of the DMN^40^. Though, in contrast to the studies reporting differences, participants in Scardua-Silva’s study were non-clinical (i.e. not seeking health care for PCC symptoms), indicating that clinical populations might differ from non-clinical in brain connectivity.

Our rs-FMRI results revealed increased functional connectivity in the precuneus, in clusters both overlapping (in the right hemisphere) and non-overlapping (in the left hemisphere) with the DMN. Notably, the precuneus is a central hub within the DMN and plays a key role in integrating information for complex cognitive functions such as visual processing, memory retrieval, and attentional shifting—functions frequently reported impaired in PCC following a mild infection^4^. As we found no significant relations between patients functional connectivity and neuropsychological test performance, it limits conclusions about the relationship between brain function and cognitive performance status. Though, a general challenge in neuropsychological assessment is the considerable variability in test scores across the general population. Consequently, average scores may be interpreted as normal despite disease-related cognitive impairment in individuals or groups with high premorbid cognitive functioning. Since we only assessed cognitive performance post-infection, any decline from premorbid levels may therefore not have been captured numerically in our well-educated patient group, potentially weakening correlations and reducing statistical power. In a larger study conducted by our research team involving 134 PCC patients with initial mild infection at our clinic, we identified significant deficits in visual attention, processing speed, and learning/memory functions (submitted manuscript). Findings from additional studies similarly demonstrate enduring neurocognitive impairments, particularly in the areas of attention, memory, and processing speed^41,42^. This further highlights the potential relevance of the precuneus in PCC-related cognitive symptoms, given its functional overlap with many of affected domains. The altered connectivity in the precuneus may reflect disrupted integration between the DMN and other cognitive systems, but as the current study did not detect connectivity changes in other brain networks or direct correlations with cognitive performance, this interpretation is speculative and needs further investigation.

Interestingly, many of the identified brain regions showing alterations in functional connectivity in PCC-patients are involved in the brains visual processing, including the left fusiform gyrus, left inferior temporal gyrus, left middle occipital gyrus and right temporal pole. Also, patients were slower than controls across visually demanding neuropsychological tests despite differences in test performance being non-significant between groups. While this study did not directly assess visual function in patients, a previous study conducted by our group showed a high prevalence of visual impairments in patients with PCC after mild infection, relating it to less efficient saccadic eye movement and binocular dysfunction^43^. Taken together, the altered brain connectivity observed in the present study provides further insight into the visual impairments previously reported, suggesting a potential link between disrupted functional connectivity in visual processing brain regions and visual impairment in patients with PCC. Future studies should aim to integrate functional brain imaging, visual neuropsychological assessments, and objective measures of ocular function to further explore this relationship.

The disappearance of significant group differences in DMN connectivity at rest following the cognitive task is noteworthy but difficult to interpret. The DMN is typically active during rest but also associated with self-referential thought, introspection and rumination^44,45^. Recent research has challenged the traditional view of the DMN as a purely task-negative network, as emerging evidence suggests its contribution also in focused cognitive processes requiring internal mental representations, even during low-effort cognitive tasks^46^. In this context, the increased DMN connectivity in patients before the PVT may reflect a less restorative cognitive state than in controls, possibly triggered by the anticipation of an upcoming task. The normalized DMN response post-task could therefore indicate a reduction in patients preparatory processes compared to the pre-task resting session, that is, a more efficient/normal post-task rest but could also reflect a potential scanner/task fatigue effect due to the long MRI session. However, this interpretation remains speculative and the non-significant time effect suggests small differences pre- and post-task.

Moreover, recent perspectives suggest that efficient brain network functioning manifest in the interaction/switching between task-positive and task-negative networks, why network abnormalities in certain disorders may not be confined to a single network^47^. Studying healthy individuals, Hugdahl, et al. demonstrated a normal time lag in DMN activation when transitioning from a cognitive task to rest^47^, why further DMN-delay could indicate less efficient switching between task-positive and task-negative networks. Thus, observed discrepancies in patients DMN-connectivity before and after the PVT could reflect a delayed DMN reactivation, indicating difficulties returning to their resting-state baseline (although elevated), as seen pre-task. Again, this is a highly speculative interpretation, requiring the continuous measurement of brain connectivity in the repeated switching between rest and a cognitive task, to be validated.

No significant associations were found between functional connectivity and self-ratings of fatigue and emotional symptoms. Consequently, our findings do not provide direct evidence the brain connectivity alterations observed are related to fatigue or emotional symptoms, in contrast to previous studies linking functional brain changes with PCC-fatigue severity^20,24^, and with anxiety and depression symptoms by themselves^48^. Nevertheless, the high fatigue scores observed in patients remain unexplained. A commonly proposed cause of fatigue in PCC is orthostatic intolerance^49^, but as we excluded patients with circulatory and cardiological conditions and participants were examined while lying down, orthostatic intolerance cannot account for the extent of fatigue in our cohort. Rather, it is possible that the lack of correlations between patients functional connectivity and fatigue were affected by the relatively low variability in fatigue ratings, making potential relations hard to detect. Importantly, both the insula and the precuneus, brain structures showing elevated functional connectivity in patients, have been pointed out as some of the central nodes in a recently proposed transdiagnostic brain-fatigue network^50^. Also, altered connectivity of the DMN has been highlighted in other conditions characterized by fatigue^50^, why we consider the investigation of PCC-related brain connectivity and its association with fatigue to be a particularly relevant focus of future research.

A potential weakness of the study is its statistical power, affecting the probability to discover true differences between groups. For example, a significant time effect was first seen in the patient group (p-value 0.05), which later diminished after controlling for multiple comparisons (p-value set to 0.025). Therefore, the difference in patients rs-functional connectivity before and after the cognitive task is uncertain. The same is true for the lack of statistically significant associations to behavioural data. In this small sample, the absence of statistically significant associations between fMRI and behavioural data should not be interpreted as evidence of no relationship, but rather as inconclusive findings due to limited statistical power or measurement sensitivity. Furthermore, information about the control groups SARS-CoV-2 history would have facilitated group infection comparison, but as testing for flu-like symptoms was not advocated in Sweden at the time of control recruitment, it was impossible to collect prior infection status in controls. Instead, we asked controls about PCC-symptoms making sure they did not have PCC. This implies that the difference between patients and controls may be smaller than it would have been had we been able to recruit control participants without prior infection. However, this was not feasible in Sweden, as the pandemic affected virtually the entire population due to the relatively liberal quarantine restrictions in place. Another limitation concerns the higher educational level observed in the control group, which may have influenced group differences in resting-state network properties and performance on neuropsychological measures. However, the groups were closely matched on estimated premorbid cognitive ability, as reflected in their comparable scores on the WAIS-IV Matrix Reasoning subtest. Because Matrix Reasoning typically shows strong correlations with general intelligence^51^, these findings suggest that premorbid cognitive functioning was similar across groups. The cross-sectional study design limits our group comparison to one time point, without assessing brain functioning/connectivity prior to infection and development of PCC. Longitudinal studies are needed to follow the brain function in the patient group over time, and to investigate if observed alterations in brain activity remain persistent. Strengths of this study include confirmed infection and well-defined PCC-diagnosis in patients, with a physician confirming the diagnosis after medical assessment. Also, the study sample consisted of mostly women with a mean age of 44 years, reflecting a group with the highest risk to develop PCC^52^. Third, we excluded with premorbid attention disorders, such as ADHD and/or other neuropsychiatric conditions, to minimize the risk that preinfection conditions affected the results.

In conclusion, our findings demonstrate that patients with cognitive complaints and PCC exhibit elevated functional connectivity in brain regions associated with the default mode network nearly 3 years following the acute infection. In this cohort of patients, neuropsychological test performance, self-reported fatigue, anxiety, and depression did not show direct associations with the observed alterations in functional connectivity. Nevertheless, patients´ self-perceived fatigue remained elevated. These subtle yet long-term brain alterations in individuals reporting long-standing cognitive symptoms and fatigue—despite having experienced only a mild initial infection—underscore the need for continued research on brain function in this patient group. Such investigations are essential to develop tailored rehabilitation for individuals with PCC with cognitive symptoms.

## Supplementary Information

Below is the link to the electronic supplementary material.

Supplementary Material 1

Supplementary Material 2

## Data Availability

The data that support the findings of this study are available on reasonable request from the corresponding author. The data are not publicly available due to ethics legislation in Sweden.

## Declarations

### Permission to reproduce material from other sources

The contribution is all the author’s original work.
