# Supplementary figures and images for: Brain connectivity and its relation to cognitive function in patients with post-COVID 19 condition after mild infection
Source: Sci Rep. 2026 Mar 3;16:8152. doi: 10.1038/s41598-026-41665-2 (PMC12960797; doi:10.1038/s41598-026-41665-2)

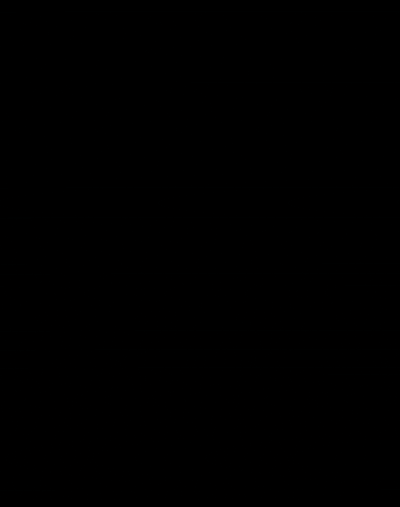

Supplement: Supplementary file 1 — Supplementary Material 1 [file 41598_2026_41665_MOESM1_ESM.gif]
