# Supplementary material for: Brain connectivity and its relation to cognitive function in patients with post-COVID 19 condition after mild infection
Source: Sci Rep. 2026 Mar 3;16:8152. doi: 10.1038/s41598-026-41665-2 (PMC12960797; doi:10.1038/s41598-026-41665-2)
Supplement: Supplementary file 2 — Supplementary Material 2 [file 41598_2026_41665_MOESM2_ESM.docx]

**Supplementary**

**Supplementary Table 1**. Description of neuropsychological tests

| **Neuropsychological test** | **Description** |
| --- | --- |
| Buschke Selective Reminding Test | Measures verbal learning and verbal memory as participants are presented to a list-learning procedure over multiple trials. The number of recalled words during the initial 12 trials and after a 30-minute delay were used as outcome variables. Higher scores indicate better performance. |
| D-KEFS Color Word | Measures executive function and inhibition of overlearned verbal responses to generate a conflicting response. Only the third condition, Inhibition, was used as an outcome variable. In this condition, patients are presented with colour names printed in incongruent colours but are asked to name the colours and not the written words. Higher scores indicate poorer performance. |
| Ruff 2 & 7 | Measures speed, sustained and selective attention as participants are presented with a scanning task on time, with the instruction to mark the digits 2 and 7. Outcome variables were Automatic detection speed (sum of the hits among letters), Controlled Search Speed (sum of the hits among digits), and Automatic detection Accuracy and Controlled Search Accuracy (number of hits compared to the total possible hits). Higher scores indicate better performance. |
| WAIS-IV Coding | Coding measures processing speed as participants are instructed to transfer as many codes as quickly as possible from a coding key, on limited time. Higher scores indicate better performance.  Coding fatigability measures cognitive fatigue, were the number of codes produced in the first 30s of the test Coding are subtracted from the number of codes produced in the tests last 30s. A non-ascending score (<0) indicates cognitive fatigue. |
| WAIS-IV Matrix reasoning | Measures visual processing and organizational skills by having participants solve visual-logical problems. Higher scores indicate better performance. |

*Note.* Adapted from a submitted manuscript by our research group to Scientific Reports, ID 84de0c45-3f71-4269-a126-50b432e309bc

**Supplementary Figure 1.** Dynamic presentation of brain clusters correlating significantly higher to IC for patient group compared to control group.
